# Gender differences in hepatocellular cancer: disparities in nonalcoholic fatty liver disease/steatohepatitis and liver transplantation

**DOI:** 10.20517/2394-5079.2018.87

**Published:** 2018-10-18

**Authors:** Eric M. Wu, Linda L. Wong, Brenda Y. Hernandez, Jun-Fang Ji, Wei Jia, Sandi A. Kwee, Sumodh Kalathil

**Affiliations:** 1 Department of Surgery, University of Hawaii, John A. Burns School of Medicine, Hawaii, 96813, USA.; 2 Department of Medicine, University of Hawaii, John A. Burns School of Medicine, Hawaii, 96813, USA.; 3 Cancer Center, University of Hawaii, Hawaii, 96813, USA.; 4 Life Sciences Institute, Zhejiang University, Hangzhou 310058, China.

**Keywords:** Hepatocellular carcinoma, non-alcoholic fatty liver disease, non-alcoholic steatohepatitis, gender, transplant

## Abstract

**Aim:**

Worldwide, hepatocellular cancer (HCC) is the fourth leading cause of cancer death and occurs 3 times more commonly in males than females. Current surveillance practices do not fully address gender differences in HCC.

**Methods:**

Clinical characteristics and survival were compared between males and females using a prospectively collected database of HCC patients.

**Results:**

In a cohort of 1206 patients, 307 (25%) were female who presented with older age, more non-alcoholic fatty liver disease/steatohepatitis (NAFLD/NASH), family history of HCC, and hypertension. Males (75%) were more likely to use alcohol and cigarettes. Females were more likely to undergo HCC surveillance, have smaller tumor size at diagnosis, and less vascular involvement. Males who met Milan criteria were more likely to undergo liver transplant than women who met the criteria. Median/mean survival was similar between the genders. Multivariate analysis showed that NAFLD/NASH was predictive of mortality for both males and females, age and smoking were predictive of mortality for males, and transplant was predictive of survival for males.

**Conclusion:**

Gender differences in HCC appear related to both behavioral risk factors and biologic factors. Older females with HCC have more NAFLD/NASH and may be overlooked by current surveillance guidelines. These gender disparities may lend support to future studies of gender-based HCC screening.

## INTRODUCTION

Hepatocellular cancer (HCC) is the fourth leading cause of cancer death worldwide and approximately 841,000 new cases are diagnosed annually^[1]^. In the US, HCC is one of the few cancers that is increasing in both incidence and death^[2]^. Viral hepatitis, a major risk factor for HCC, has declined in relative importance as vaccination for hepatitis B has become almost routine and treatments for chronic hepatitis B and C virus (HBV and HCV) have improved. Concomitantly, metabolic conditions and fat-related liver disease have become increasingly prominent risk factors^[3,4]^. Although HCC is historically more common in Asian/Pacific Islanders and males, the incidence is increasing in Hispanics, Blacks, and females^[5]^.

Although current guidelines on HCC surveillance from leading professional organizations focus on high-risk populations, there is no consensus as to the optimal surveillance in those with non-alcoholic fatty liver disease/steatohepatitis (NAFLD/NASH). A large part of the problem is difficulty in identification of the population at risk as many of these patients have undiagnosed NAFLD/NASH. They are typically followed by only primary care physicians for diabetes or hyperlipidemia or perhaps followed by a hematologist for unexplained thrombocytopenia.

HCC predominantly affects males with incidence two to four times more common in males than females^[6]^. The reasons for this gender disparity are complex and may stem from differences in behavioral risk factors, metabolic factors, tumor biology, and treatments received. Of note, there are gender differences in metabolic factors and NAFLD/NASH that may be helpful in developing guidelines for HCC surveillance. Obesity is more prevalent in females than males with currently 38% of US females being obese^[7]^. Type II diabetes mellitus is more common in males than females but females are more likely to have cardiovascular disease, myocardial infarction and cerebrovascular accidents^[8]^. Males are overall more likely than females to have NAFLD/NASH, however, after the age of 60 years, females are much more likely to have NAFLD/NASH^[9,10]^. Estrogen is believed to have a protective role in the development of HCC as differences in subtypes of estrogen receptors expressed in males vs. female have been shown to contribute to the progression of HCV related HCC^[11]^. As fat related liver diseases increasingly emerge as the most common cause of chronic liver disease, it is crucial that the relationship between fatty liver disease and HCC is fully explored.

The purpose of this study is to comprehensively evaluate gender differences in a large cohort of HCC patients-to better define populations at risk for evaluation in future surveillance studies.

## METHODS

### Study participants

A retrospective analysis was conducted using de-identified clinical and outcome data from 1206 HCC cases diagnosed between 1993 and 2017 by a group of physicians associated with a medical center having the only liver transplant program in Hawaii, as well as the only referral center for liver disease for the American territories of the Pacific Basin and other Pacific Island Nations, including Samoa, Guam, Saipan, Micronesia and the Marshall Islands. This clinic and the transplant center were initially affiliated with Hawaii Medical Center-East (formerly St. Francis Medical Center) and after 2012, with the Queens Medical Center. About 60%−70% of HCC cases from the State of Hawaii are seen in this center. Other patients in this cohort were foreign nationals from Asian countries, including China, Japan, Korea, and the Philippines, who pursued medical care in the US. This study was approved by the University of Hawaii Institutional Review Board.

The diagnosis of HCC was confirmed histologically (percutaneous biopsy or at surgery) or with a combination of imaging and alpha-fetoprotein (AFP). Patients diagnosed in the first decade were included if they had a history of chronic liver disease and a liver mass that was least 2 cm in size and seen on two imaging studies (ultrasound, CT scan or MRI) and one of the following: (1) vascular blush seen on CT scan or MRI; (2) AFP > 200 ng/mL; or (3) arteriogram confirming the tumor. More recently, the diagnosis of HCC was verified with only imaging if a contrast-enhanced study (dynamic CT or MRI) showed typical arterial enhancement with “washout” in the venous phase as described by the American Association for the Study of Liver Disease guidelines^[12,13]^.

### Data collection

Information on demographics, medical history, laboratory results, tumor characteristics, treatment, and survival was obtained from medical records. Demographic data included age, sex, birthplace, and the patient’s self-reported ethnicity. Ethnicity was then categorized as “White”, “Asian” (including Filipinos), or “Pacific Islander”. Patients who did not fit into one of these categories or were of mixed ethnicity were subsequently classified as “Mixed”. Patients of mixed race with 50% Pacific Islander ethnicity were categorized as “Pacific Islander”. Risk factor information that was collected included: diabetes mellitus, hyperlipidemia, smoking, viral HBV and HCV, alcohol abuse (defined as greater than two alcoholic beverages daily for at least ten years), and other chronic liver diseases. Information was based on available medical records and interviews, without use of a structured questionnaire. Patients who did not report hyperlipidemia but had a lipid-lowering agent on their current medication list were also classified as having hyperlipidemia. Measured height and weight were used to determine body mass index (BMI). Obesity was defined as BMI ≥ 30. Patients with no viral, alcohol risk factors or other known liver disease were categorized as NAFLD if documented by imaging or liver biopsy showing steatosis. Those with no viral or alcohol risk factors were classified as NASH if imaging or biopsy showed cirrhosis.

Laboratory data collected (within 2 weeks of initial visit) included bilirubin, albumin, prothrombin time, creatinine, alanine aminotransferase (ALT), aspartate aminotransferase (AST), platelet count and AFP. Model for end-stage liver disease (MELD) score and fibrosis markers, fibrosis-4 score (FIB4) and AST/platelet ratio index (APRI) were also calculated. The size and number of the tumor(s) were used to determine the tumor node metastases stage according to the American Joint Commission on Cancer staging manual^[14]^. Vascular invasion was only reported as macrovascular invasion based on imaging studies as not all patients had sufficient tissue specimen to provide useful analysis of microvascular invasion.

The proportion of patients with HCC detected with surveillance was noted. Although current guidelines recommend surveillance of patients with cirrhosis and chronic HBV or HCV with AFP and liver ultrasound every six months, there was no uniform screening protocol used in the cohort. Referring physicians used a combination of AFP and/or imaging (ultrasound, CT scan or MRI) at variable intervals. HCC was deemed to be found on “screening” if the referring physician stated that screening was done and/or the patient had a previous imaging study from three to twelve months prior. HCC not found on screening was either diagnosed with symptoms (pain, abdominal mass, weight loss, jaundice) or asymptomatically with imaging done for unrelated reasons and incidental discovery of a liver mass.

### Treatments

Treatments included liver resection, transplantation, loco-regional therapies (including radiofrequency ablation, cryosurgery, transarterial chemoembolization, and percutaneous ethanol injection) and systemic therapies. Liver resection was considered in Child’s A patients and early Child’s B patients (Childs Turcotte Pugh score of 7, without any evidence of ascites or encephalopathy). Liver transplantations were considered in patients who had unresectable HCC but met Milan criteria (single tumor less than 5 cm or 2–3 tumors, each less than 3 cm). Liver transplantation was also considered in patients who underwent resection but had recurrence of HCC more than six months after surgery, provided the recurrent tumor met Milan criteria and there was no disease progression while awaiting transplant. Since 2007, liver transplantation was considered in patients who met UCSF criteria (single tumors less than 6.5 cm, 2–4 tumors with total diameters less than 8.5 cm) provided that their HCC had been downstaged to meet Milan criteria with locoregional therapy and AFP was less than 1000 ng/dL. All liver resections and transplantation were performed by members of our surgical group. The majority of patients on the transplant list underwent locoregional therapy as a bridge to transplant.

Patients were followed with imaging every 3 months after surgery or locoregional therapies for the first year and subsequently every 4–6 months. Most of these patients were followed by the physicians involved in the initial treatment, so follow up and survival were carefully monitored. Deaths were confirmed using the Social Security Death Index and local newspaper obituaries.

### Statistical analysis

All analyses were performed using Excel and SPSS statistical software. Categorical variables were analyzed using chi-square analysis and Students t-test was used to determine significant differences in numerical values. Univariate and multivariate logistic regression were used to determine factors that were associated with receiving transplantation. Factors included gender, age < 60 years, hypertension, NAFLD/NASH, family history of HCC, alcohol, smoking, whether they had a screenable disease, obesity, education, HBV, HCV and race. Multivariate Cox proportional hazards regression was used to determine factors that were associated with survival in males and females separately.

## RESULTS

### Overall cohort

In this cohort of 1206 patients, 899 (74.5%) were male and mean age overall was 62.7 years with 41.6% of patients being 65 years or older. Ethnic distribution was as follows: Asian (59.5%), White (20.2%), Pacific Islander (15.4%), Mixed (2.2%), Hispanic (1.8%) and Black (0.9%). HBV surface Ag was positive in 26.2% and another 10.9% were positive for HBV core Ab but negative for surface Ag. The overall incidence of HCV antibody was 40.8%. About 11% of patients in the cohort had no viral or alcohol risk factors and had documented NAFLD or NASH on imaging or biopsy.

### Differences between males and females

#### Demographics and risk factors

Differences in demographics and risk factors are summarized in Table 1. Females developed HCC at a significantly older age (66.0 years vs. 61.6 years, P < 0.001) with a larger proportion greater than 65 years old (53.4% vs. 27.6%). Females trended toward having less incidence of HBV surface Ag, core Ab and HCV positivity however this was not statistically significant. A higher proportion of males were coinfected with both HCV and HBV (7.0% vs. 3.6%). Overall, females were more often screened for HCC (29.3% vs. 22.7%, P = 0.02) and had greater rates of NAFLD/NASH (21.5% vs. 7.2%, P < 0.0001) and hypertension (67.2% vs. 54.8%, P = 0.0007).Elderly females (≥ 65 years) were more likely than elderly males to have a NAFLD/NASH related HCC (28.0% vs. 14.8%, P = 0.0006). Furthermore, elderly females were also more likely to have NAFLD/NASH than younger females, as 46 of 164 older women had NAFLD/NASH compared to 20 of 143 younger women who had NAFLD/NASH (28% vs. 14%, P = 0.003). Females with a screenable disease (based on existing practice guidelines) were also more likely to undergo HCC screening than men with screenable disease (41.6% vs. 28.7%, P = 0.0005). Males were more likely to smoke (68.4% vs. 38%, P = 0.0001) and drink alcohol (52.9% vs. 12.1%, P = 0.0001). Females were more likely to have a family history of HCC (8.8% vs. 5.3%, P = 0.04). There was no significant difference in educational attainment, viral hepatitis rates, obesity, diabetes and hyperlipidemia.

#### Laboratory data

Table 2 summarizes differences in laboratory studies. Males had a higher rate of normal AFP (40.6% vs. 31.7%, P = 0.0064), higher mean bilirubin (1.8 vs. 1.4, P = 0.03), creatinine (1.09 vs. 0.95, P = 0.01), AST (90.9 vs. 72.4, P = 0.001) and ALT (73.3 vs. 52.4, P < 0.001). The MELD score was also higher in males (10.8 vs. 10.0, P = 0.007). There were no significant differences in mean AFP, albumin, platelets, cholesterol, triglycerides, APRI or FIB4 score between males and females.

#### Tumor characteristics and treatments

Differences in tumor characteristics and treatments are summarized in Table 3. Males had a larger mean tumor size (6.2 vs. 5.3, P = 0.003), with more tumors > 5 cm (43.4% vs. 34.5%, P = 0.007). Females had more tumors that met Milan criteria (47.9% vs. 40%, P = 0.05). HCC in males more often involved major vessels (12% vs. 7.5%, P = 0.03). There were no significant differences in the percentage of patients that presented with a single tumor or the receipt of resection or transplant. However, among the patients that met Milan criteria, men were more likely than women to receive transplant (29.6% vs. 10.9%, P < 0.0001).

#### Factors associated with transplantation

Table 4 summarizes differences in factors associated with transplantation. Univariate analysis determined that age < 65 years, presence of screenable disease and having HCV were associated with receiving transplant, while hypertension, having high school or less education and being of Asian or Pacific Islander ethnicity relative to Caucasian ethnicity were associated with lower rates of transplant. Multivariate logistic regression analysis determined that age < 60 years, presence of NAFLD/NASH and having a screenable disease were associated with transplantation. Factors not significantly associated with transplantation included sex, hypertension, educational attainment, HCV infection, or race.

#### Survival

**Figure 1.**
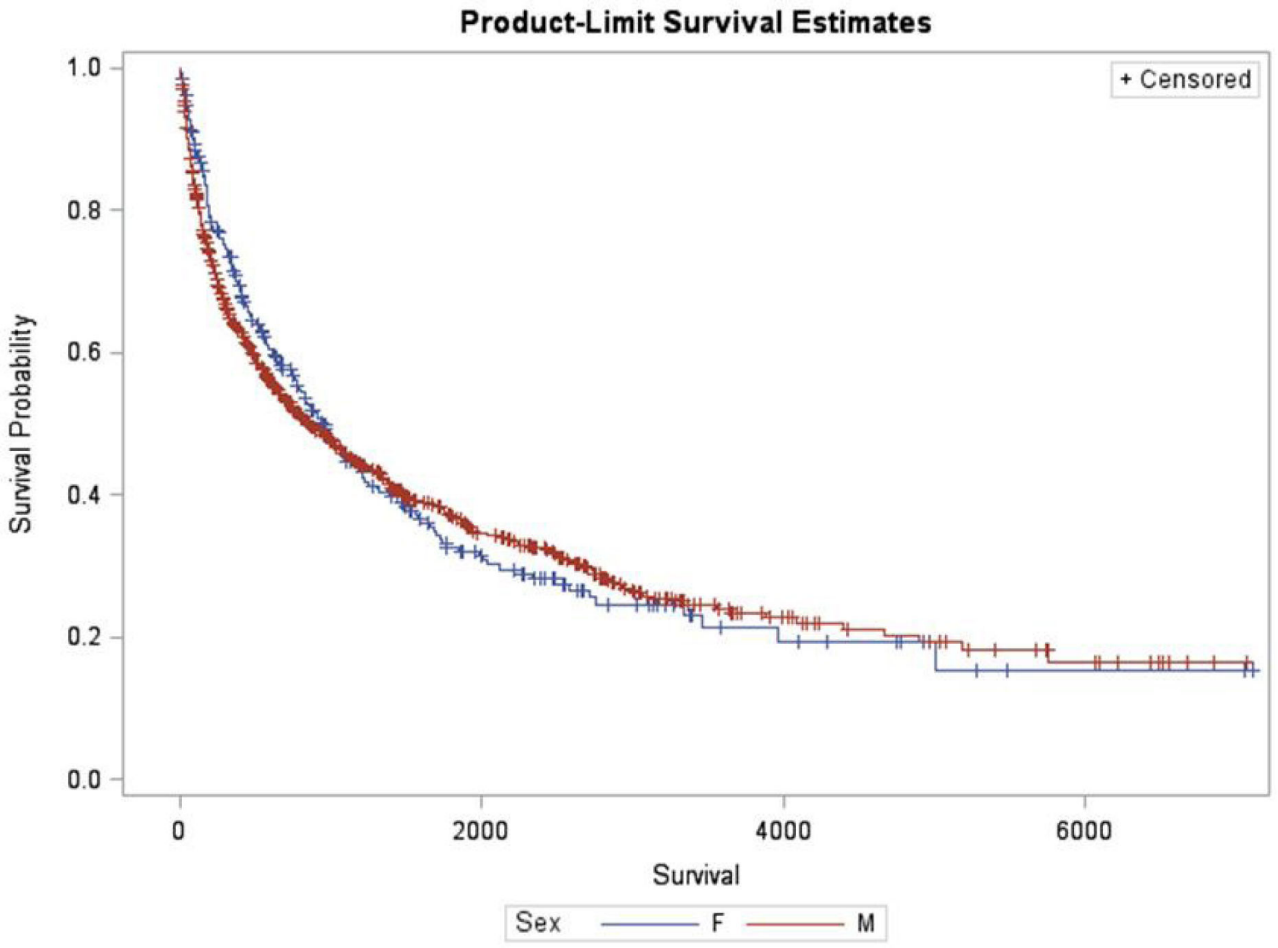
Kaplan-Meier survival: comparison of males *vs*. females. Survival is measured in days

Survival outcomes are displayed in Figure 1. There was no significant difference in survival between males and females by the log-rank test (P = 0.69, see Figure 1). Table 5 summarizes the independent predictors of death. Multivariate Cox proportional hazards regression showed that NAFLD/NASH was a predictor of death in both males and females. Smoking and number of tumors were predictors of death while age less than 65 years, a family history of HCC and undergoing liver transplant were predictive of survival in males.

## DISCUSSION

Gender differences in HBV and HCV may partially explain the male predominance of HCC, however geographic variations, hormonal changes, environmental/behavioral risk factors and compliance with antiviral therapies may further influence these differences. Males are more likely to acquire HBV and HCV, develop chronic hepatitis, cirrhosis and HCC. This progression may be related to lower seroconversion after HBV vaccination compared to females, as well as androgen related upregulation of viral production and inflammation^[15,16]^. For both HBV and HCV, there is evidence that female gender confers a protective effect against HCC as estrogen decreases IL-6 mediated hepatic inflammation and viral production^[17–19]^. While this study cannot make definitive conclusions without knowledge of all HBV and HCV patients at risk, females trended toward having less HBV and HCV although this was not statistically significant. Although a recent meta-analysis reported that co-infection with HBV and HCV did not increase HCC risk^[20]^, our study did show that males in the cohort were more likely to be coinfected.

Behavioral risk factors such as smoking and alcohol are known independent risk factors for HCC^[21]^. Alcohol damages the liver through oxidative stress and inflammation that results in a spectrum of fatty changes from reversible damage to cirrhosis. In the US, HCC attributed to alcohol usage is more common in males (27.8%) than females (15.4%)^[22]^. In our study, a larger proportion of males had significant alcohol usage compared to females, although we did not exactly quantify the amount of alcohol used nor account for past vs. current alcohol use. Smoking has been shown to increase both the incidence and mortality of HCC, and males in our study were more likely to smoke. Smoking was also an independent predictor of mortality in males in our study, while alcohol did not affect mortality in either gender. Despite the inability to determine dose effects of alcohol and smoking, our data confirms that there are gender differences in behavioral risk factors for HCC.

Gender differences in metabolic risk factors for HCC are important as NAFLD is currently the most common chronic liver disease in western industrialized countries^[23]^. Differences in adipocyte metabolism may contribute to the gender disparity in HCC^[24]^. Visceral adiposity, more common in males, has been shown to induce a pro-inflammatory state that could increase risk of fibrosis relative to females, who may be protected by estrogen^[25]^. This protection may be lost in postmenopausal women, where NAFLD rates have been shown to increase with age relative to men^[26]^. The relative increase in visceral adiposity in males may help explain the gender disparity in HCC, as one study showed an association of BMI with HCC risk only in males^[27]^. Females in our study had higher rates of NAFLD/NASH than males, with older women having significantly higher rates of NAFLD/NASH than younger women and older men. Our study showed that NASH associated HCC disproportionately affected older women but a longitudinal study of a large population of NASH patients would be necessary to validate this.

Surveillance has been shown to decrease mortality from HCC in multiple retrospective studies^[27]^. However, data on gender differences in HCC surveillance have been inconsistent^[28–31]^ and gender disparities in surveillance rates may impact prognosis^[32]^. In this study, females with a screenable disease were more likely to have HCC identified with surveillance, but this did not impact their survival. One possible explanation is that females overall were less likely to have a known screenable disease and more likely to have a fat-related liver disease, an HCC risk factor for which there are no established screening guidelines unless cirrhosis is present. Furthermore, HCC attributed to NAFLD has been shown to frequently develop in non-cirrhotic livers^[33]^, decreasing the likelihood of early tumor detection. Despite a higher rate of HCC detection through surveillance, a considerable proportion of females at risk for HCC may be overlooked with regards to screening.

Gender disparities in transplantation are well described in the literature, with males tending to undergo transplantation more than females^[33]^. Gender disparity may result from the fact that males more commonly present with the leading indications for transplant (alcohol and HCV induced cirrhosis) and are more likely than females to have early-referral to a transplant center^[34,35]^ while females may have lower MELD scores due to relatively less muscle mass and creatinine^[36,37]^ and finally, donor-recipient organ size mismatch^[38,39]^. In our study, females trended towards meeting Milan criteria and males trended towards having more liver transplants. If only those patients who met Milan criteria were considered as potential transplant candidates, males were significantly more likely to undergo liver transplant. In the multivariate analysis, the significant factors for receiving a liver transplant were age, the presence of NAFLD/NASH, and presence of screenable disease. This may suggest that efforts to improve transplant rates should be directed towards better screening for patients with NAFLD and NASH.

This study did not show a survival difference between the genders but contained a more detailed risk factor analysis than previous studies^[40]^ which demonstrated that NAFLD/NASH was the only factor associated with mortality in both genders. Receiving a liver transplant was associated with improved survival in males but not females. While one would expect that liver transplant would improve survival in both genders, perhaps the fewer numbers of females undergoing transplant in our cohort made the overall survival benefit in females less apparent. Females were less likely to receive liver transplants despite being more likely to meet Milan criteria, have NASH/NAFLD and have HCC found with surveillance. Clearly there are other reasons that contribute to getting a liver transplant that could not be delineated in this study which may include insurance issues, substance abuse, comorbidities and potentially cultural issues in a predominantly Asian population. Although we cannot determine causation, our data suggests that NAFLD/NASH may lead to increased mortality due to decreased surveillance in this population and less opportunity for curative therapies. Some of these patients were likely diagnosed with NAFLD but were not followed closely and thus, were allowed to progress to HCC.

A limitation of this study was that it consisted of a single-center retrospective study in a relatively isolated population. Some of the differences in risk factors and treatment by gender might have been affected by ethnicity, as well as cultural and language barriers because more than a third of the patients were born outside the US. It was also difficult to truly separate all of the risk factors to determine causality as many patients had combinations of risk factors and dose/time/severity dependent factors such as alcohol usage, smoking, obesity and diabetes. We also did not collect data on whether a patient was pre or post-menopausal and whether there was any usage of hormone replacement therapy so it was difficult to make conclusions about the contribution of sex steroids on the development of HCC. Finally, we may have underestimated the NAFLD/NASH group, as there were patients with no viral risk factors or alcohol usage, but with metabolic risk factors and not enough information on imaging or biopsy to categorize them as NAFLD/NASH. Despite these limitations, the strengths of our study include a robust sample size, diverse study population, and detailed risk factor data that may not be available in administrative or national cancer databases. Furthermore, because we are Hawaii’s only dedicated liver center that sees nearly 70% of Hawaii’s HCC cases, we believe that this study gave an accurate view of a state with a high burden of HCC.

We have shown that there are distinct gender differences in behavioral and metabolic risk factors as well as access to liver transplantation that disproportionately affects certain subgroups with regards to HCC. Older women with HCC appear to have higher rates of underlying NAFLD/NASH but this population may be overlooked by current surveillance guidelines, thus losing a valuable opportunity for early tumor detection and treatment. The epidemic of NAFLD/NASH may potentially increase HCC disproportionately in older females but further studies will be needed to validate this. Future efforts should be directed towards better identification of NAFLD/NASH in this population and how to effectively survey these patients for HCC.

## Figures and Tables

**Table 1.** Demographics and risk factors: comparison between females and males

	Females (*n* = 307)	Males (*n* = 899)	*P*-value
Mean age in years (SD)	66.0 (11.3)	61.6 (11.3)	< 0.001
Age ≥ 65 years	164 (53.4%)	338 (27.6%)	< 0.0001
Race			0.002
Asian	213 (69.4%)	505 (56.2%)	
Black	0	9 (1%)	
Hispanic	4 (1.3%)	18 (2.0%)	
Mixed	8 (2.6%)	19 (2.1%)	
Pacific Islander	39 (12.7%)	147 (16.4%)	
White	43 (14%)	201 (22.4%)	
Finished high school	149/191 (78%)	494/606 (87.5%)	0.29
Hepatitis B sAg+	69/304 (22.7%)	248/896 (27.7%)	0.10
Hepatitis B coreAb+	27/304 (8.9%)	104/896 (11.6%)	0.20
HCV+	112/304 (36.9%)	382/895 (42.5%)	0.08
Alcohol use	37/306 (12.1%)	474/896 (52.9%)	0.0001
Screenable disease	209/307 (68.1%)	705/899 (78.4%)	0.0003
HCC found on surveillance^*^	87/209 (41.6%)	202/705 (28.7%)	0.0005
NAFLD/NASH	66 (21.5%)	65 (7.2%)	< 0.0001
NAFLD/NASH(age ≥ 65)	46/164 (28.0%)	50/338 (14.8%)	0.0006
Mean BMI	26.3 (5.86)	27.0 (5.32)	0.05
Obesity (BMI ≥ 30)	61 (19.9%)	176 (19.6%)	0.93
Smoking history	114/300 (38%)	607/888 (68.4%)	0.0001
Current Smoker	24/300 (8%)	109/888 (12.3%)	0.04
Diabetes	116 (37.8%)	289 (32.9%)	0.21
Hyperlipidemia	72/304 (23.7%)	203/873 (23.3%)	0.88
Hypertension	160/238 (67.2%)	396/726 (54.8%)	0.0007
Family History of HCC	27 (8.8%)	48 (5.3%)	0.04

* Includes only those with a screenable disease. HCV: hepatitis C; HCC: hepatocellular cancer; NAFLD: non-alcoholic fatty liver disease; NASH: non-alcoholic steatohepatitis; BMI: body mass index

**Table 2.** Laboratory data: comparison between females and males

	Females (*n* = 307)	Males (*n* = 899)	*P*-value
Normal AFP	97/306 (31.7%)	363/895 (40.6%)	0.0064
Mean AFP (ng/mL)	14,962 (67797)	13,257 (61588)	0.68
Mean bilirubin (mg/dL)	1.4 (1.97)	1.8 (2.74)	0.03
Mean albumin (g/dL)	3.5 (0.66)	3.5 (0.71)	0.44
Platelets (1O^3^/mm^3^)	162.6(99.8)	169.6 (98.4)	0.29
Creatinine (mg/dL)	0.95 (0.88)	1.09 (0.84)	0.01
AST(U/L)	72.4 (61.8)	90.9 (84.6)	0.001
ALT (U/L)	52.4 (43.4)	73.3 (61.7)	< 0.001
Cholesterol (mg/dL)	163.3 (53.5)	163.8 (42.6)	0.94
Triglyceride (mg/dL)	104.7 (43.9)	123.1 (74.8)	0.81
MELC	10.0 (4.36)	10.8 (4.58)	0.007
APRI	1.2 (2.12)	1.1 (1.68)	0.35
FIB4	5.7 (5.09)	5.3 (4.36)	0.21

AFP: alpha-fetoprotein; AST: aspartate aminotransferase; ALT: alanine aminotransferase; MELD: model for end-stage liver disease; APRI: AST/platelet ratio index; FIB4: fibrosis-4 score

**Table 3.** Tumor characteristics and treatments: comparison between females and males

	Females (*n* = 307)	Males (*n* = 899)	*P*-value
Mean tumor size in cm (SD)	5.3 (4.02)	6.2 (4.58)	0.003
Tumor > 5 cm	106 (34.5%)	496 (43.4%)	0.007
Single tumor	213 (69.4%)	588 (65.4%)	0.21
Tumors met Milan criteria	147 (47.9%)	260 (40%)	0.05
Tumor rupture	14 (4.5%)	35 (3.9%)	0.62
Major vascular invasion	23 (7.5%)	108 (12%)	0.03
Liver resection	68 (22.1%)	168 (18.7%)	0.30
Liver transplantation	16 (5.2%)	77 (8.6%)	0.06
%Transplant/met Milan criteria	16/147 (10.9%)	77/260 (29.6%)	< 0.0001

**Table 4.** Odds-ratios of factors associated with transplantation (modeled using logistic regression)

Factor	Univariate odds ratio (95% CI)	Multivariate odds ratio (95% CI)
Sex (males *vs*. females)	1.71 (0.98–2.97)	1.48 (0.76–2.88)
Age (< 65 *vs*. ≥ 65)	9.84 (4.052–21.45)	10.21 (3.88–26.99)
Tumor size	0.81 (0.49–1.33)	
Hypertension	0.61 (0.3–0.96)	0.92 (0.55–1.55)
NAFLD/NASH	0.66 (0.30–1.45)	4.14 (1.42–12.05)
Family history of HCC	1.25 (0.56–2.79)	
Alcohol use	0.93 (0.60–1.43)	
Smoking	0.71 (0.47–1.10)	
Presence of screenable disease	9.91 (3.10–31.61)	11.52 (3.03–43.76)
Obesity (BMI 30+)	1.13 (0.68–1.90)	
Education *(*≤13 *vs. >* 13 years)	0.51 (0.33–0.79)	0.63 (0.38–1.05)
Hepatitis B positive	0.91 (0.59–1.42)	
Hepatitis C positive	2.34 (1.52–3.60)	1.55 (0.88–2.76)
Race (reference = White)		
Asian	0.58 (0.36–0.94)	1.09 (0.88–2.76)
Hispanic	0.77 (0.17–3.46)	0.56 (0.07–4.60)
Mixed	0.96 (0.27–3.40)	0.36 (0.04–2.93)
Pacific Islander	0.39 (0.18–0.85)	0.58 (0.25–1.36)

HCC: hepatocellular cancer; NAFLD: non-alcoholic fatty liver disease; NASH: non-alcoholic steatohepatitis; BMI: body mass index

**Table 5.** Factors predictive of death (Cox regression) by gender

Parameter	Hazard ratio (95%CI) males	*P*-value	Hazard ratio (95% CI) females	*P*-value
Age (< 65 vs. ≥ 65 years)	0.65 (0.47–0.90)	0.009	0.78 (0.47–1.30)	0.35
Liver transplant	0.47 (0.33–0.68)	< 0.0001	0.66 (0.28–1.53)	0.34
Number of tumors	1.20 (1.06–1.36)	0.003	1.14 (0.78–1.70)	0.48
Hypertension	0.88 (0.67–1.16)	0.38	0.99 (0.58–1.68)	0.97
NAFLD/NASH	2.02 (1.22–3.33)	0.006	2.29 (1.20–4.35)	0.01
Family history of HCC	0.57 (0.34–0.97)	0.038	0.89 (0.38–2.08)	0.78
Alcohol use	0.97 (0.73–1.30)	0.86	1.64 (0.85–3.16)	0.14
Smoking history	1.78 (1.32–2.38)	< 0.0001	1.29 (0.82–2.03)	0.27
HCC found on surveillance	1.22 (0.83–1.79)	0.31	1.31 (0.76–2.23)	0.34

HCC: hepatocellular cancer; NAFLD: non-alcoholic fatty liver disease; NASH: non-alcoholic steatohepatitis
